# Hyponatremia Presenting with Recurrent Mania

**DOI:** 10.7759/cureus.3645

**Published:** 2018-11-28

**Authors:** Sahil Parag, Eduardo D Espiridion

**Affiliations:** 1 Medical Education and Simulation, West Virginia School of Osteopathic Medicine, Lewisburg, USA; 2 Psychiatry, Frederick Memorial Hospital, Clear Spring, USA

**Keywords:** primary psychogenic polydipsia, hyponatremia, bipolar mania

## Abstract

Primary psychogenic polydipsia (PPD) is a chronic, relapsing condition in which there is a disturbance in thirst control primarily due to an underlying disorder such as a psychogenic condition. It is characterized by an increase of fluid intake along with excretion of excessive amounts of dilute urine exceeding 40 to 50 mL/kg of body weight. PPD is typically seen in patients with schizophrenic symptoms due to elevated levels of dopamine that stimulate the thirst center or in patients with a psychiatric history receiving anticholinergic drugs.

There are many reported cases of PPD related to an underlying schizophrenia disorder, but rarely is PPD seen in bipolar patients.

We herein report a case of recurrent mania in a patient from a community hospital, who presented with chronic hyponatremia due to PPD. The patient had a history of bipolar disorder type 1 and was admitted to the hospital four times within three weeks with hyponatremia and presenting symptoms of mood lability, psychomotor agitation, pressured speech, racing thoughts, sleeping disturbances, distractibility, and inflated self-esteem. These were the same circumstances and manic presentation in her subsequent medical admissions.

Due to her repeat manic presentation and consistently low sodium levels, we believe that her manic symptoms were a result of hyponatremia due to PPD. This patient serves as a unique case wherein switching medications and treating with oral sodium chloride did not prevent the manic episodes as she continues to become hyponatremic secondary to PPD. Due to the difficulty in managing and diagnosing a patient like this, case studies are helpful in studying treatment and maintenance for future cases.

## Introduction

Hyponatremia is defined as a serum sodium level below 135 mEq/L and is only found in 10% to 20% of all primary psychogenic polydipsia (PPD) patients. Symptoms are typically not evident unless there is an acute 3 to 4-L ingestion or if patients continue to drink excessively (more than 10 L daily) after reaching their limit of urine dilution and antidiuretic hormone (ADH) suppression [1-6]. An acute drop in serum sodium levels below 125 mEq/L can cause headache, nausea, confusion, delirium, and even sudden death [7]. Thus, in patients with a previously diagnosed psychiatric disease with recurrent hyponatremia, PPD must be diagnosed as one of exclusion. Other clinical investigations of diabetes insipidus, syndrome of inappropriate antidiuretic hormone secretion (SIADH), and urine osmolarity must be completed to rule out other causes [8]. Our patient’s acute serum sodium levels played a role in her recurrent manic symptoms even with medication control.

## Case presentation

A 61-year-old Caucasian female presented to a community hospital with a history of hyponatremia, arthritis, migraines, and bipolar disorder. She was admitted after coming to the emergency department (ED) on an emergency petition after her husband called the police. Her husband stated he called due to her manic symptoms and forgetting to turn the stove off. She was previously diagnosed with bipolar disorder in September 2017 for which she took divalproex 250 mg daily and quetiapine 50 mg at bedtime. At the time of the interview, the patient displayed hyperverbal pressured speech with rambling. Her thought process was goal oriented with bouts of loosening associations. She denied suicidal thoughts and use of alcohol, and she reported difficulty sleeping for which she used medical marijuana regularly. The patient appeared hypomanic, and her cognition and sensorium appeared clear.

At the time of admission, her sodium level was 129 mEq/L, with decreased hemoglobin and hematocrit. She was given olanzapine 5 mg orally at bedtime and was discharged two days later after her sodium levels and manic symptoms normalized from fluid restriction and oral sodium chloride.

Two days after discharge, the patient presented to the hospital with manic symptoms and was found to be hyponatremic. At admission, her sodium levels were 128 mEq/L. After treating her mania and restricting her fluids, her sodium level rose to 134 mEq/L. The patient had clear thoughts, speech, and cognition the following day. She stated that she was not drinking as many fluids, but, per the nurses, she was constantly requesting fluids. 

On the third day of her admission, her sodium levels fell to 128 mg/L with a urine osmolarity of 268 mOsm/kg (reference range: 275 to 300 mOsm/kg), and a suspected increase in overnight fluid consumption was noted (Table 1). She had a normal mental state, cognition, and sensorium, but was in distress about her constant fluctuating levels and wanted to leave the hospital. It appeared that she was having racing thoughts at this time and continued to discuss plans of divorcing her husband of 38 years and staying in a motel. SIADH and PPD were both considered at this time. She was further switched from divalproex and olanzapine to perphenazine and added sodium chloride tablets. She was discharged the following day.

**Table 1:** Lab values per admission BUN: blood urea nitrogen, GFR: glomerular filtration rate

Lab value (reference range)	Admission 1	Admission 2	Admission 3	Admission 4
Sodium (135-145 mEq/L)	129	128	129	128
BUN (7-20 mg/dL)	9	8	8	6
Creatine (0.5-1.1 mg/dL)	0.4	0.3	0.4	0.5
Calculated Osmolality (275-299 mOsm/L)	266	264	267	266
GFR (>60 mL/minute)	>60	>60	>60	>60
Glucose (70-99 mg/dL)	102	112	117	100
Potassium (3.5-5.0 mEq/L)	3.8	3.6	3.8	4.1
Chloride (96-106 mEq/L)	93	91	91	89

A week following her third discharge, the patient was readmitted to the hospital with similar symptoms and hyponatremia. After nephrology consultation, she was diagnosed with reset osmostat (i.e., ADH shutting down due to low sodium levels). Her continuous cycles had shown that she was unable to control her fluid intake while at home and that she was at risk for continual admittance to the ED for hyponatremia with recurrent mania.

## Discussion

In this patient, excess water intake leading to hyponatremia was thought to be a result of her initial anti-manic medication. Her resulting hyponatremia was followed by recurrent mania that did not improve upon a change in medication. The typical presentation of PPD includes an underlying schizophrenia diagnosis. This case serves as a unique example for which PPD causes secondary hyponatremia leading to recurrent mania.

The patient was initially started on divalproex in September 2017. Divalproex (valproic acid) is a fatty acid with anticonvulsant properties used in the treatment of manic symptoms of bipolar disorder. Its exact mechanism is not well understood, but it is thought to work by increasing the gamma-aminobutyric acid levels or altering the properties of voltage-gated sodium channels [9]. The suppression of voltage-gated sodium channels helps suppress repetitive neuronal firing [10]. Therefore, sodium valproate can cause SIADH-like syndrome with hyponatremia, and sodium levels must be monitored for symptoms of mania while receiving treatment. This led us to believe that the divalproex was the underlying cause of our patient’s chronic hyponatremia and, thus, recurrent manic symptoms.

Due to these side effects, the patient was switched to olanzapine on her visit to the ED in July 2018. Olanzapine is an atypical antipsychotic agent used to treat acute symptoms of mania by antagonizing the dopamine (D2) receptors in the mesolimbic pathway and the serotonin (5HT2A) receptors in the frontal cortex [11]. Unlike divalproex, olanzapine does not have the same side effect profile of SIADH and is unlikely to cause chronic hyponatremia in this patient.

Her manic symptoms remained. In response, we switched her divalproex and olanzapine to perphenazine and added sodium chloride tablets. Perphenazine is an antipsychotic that works as a D1/D2 receptor antagonist and can also control severe nausea and vomiting [12].

PPD was the likely diagnosis because she presented with hyponatremia, low random urine osmolarity, and a low blood urea nitrogen (BUN) level.

In previous case reports of patients with an underlying psychogenic disorder, switching medications have shown efficacy in treating PPD [13]. The persistence of the patient’s hyponatremia with a low BUN level even after switching medications suggests that her hyponatremia may be due to fluid overload secondary to PPD. The recurrence of her manic symptoms during these bouts further suggests that she is suffering from recurrent mania due to chronic hyponatremia. Controlling her fluid intake and maintaining her antipsychotic medication is the best way to normalize her sodium levels, thus preventing her manic presentation.

During each admission, her sodium levels, manic behavior, confusion, and speech vastly improved, primarily due to the control of her fluid consumption. Her nurses noted that the patient constantly complained of excessive thirst and frequently asked for more fluids. The repeat admissions proved that she was unable to maintain a normal balance, even after education on fluid intake.

## Conclusions

The continuation of the patient’s symptoms, hyponatremia, and low BUN levels with three medication changes helped rule out SIADH, leading us to believe that it was mainly due to PPD. PPD is a chronic condition with a relatively difficult treatment. This patient serves as a unique example, wherein the patient continues to present with manic symptoms due to her hyponatremic state. The best future management for this patient involves providing both education on the importance of fluid restriction and adequate means for her to maintain a stable sodium level while at home. This patient serves as an example of a unique case and should be considered on future differentials in order to reduce readmission rates.
